# Antibody Responses to SARS-CoV-2 mRNA Vaccines Are Detectable in Saliva

**DOI:** 10.20411/pai.v6i1.441

**Published:** 2021-06-07

**Authors:** Thomas J. Ketas, Devidas Chaturbhuj, Victor M Cruz Portillo, Erik Francomano, Encouse Golden, Sharanya Chandrasekhar, Gargi Debnath, Randy Díaz-Tapia, Anila Yasmeen, Kyle D. Kramer, Tarek Munawar, Wilhelm Leconet, Zhen Zhao, Philip J.M. Brouwer, Melissa M. Cushing, Rogier W. Sanders, Albert Cupo, Per Johan Klasse, Silvia C. Formenti, John P. Moore

**Affiliations:** 1 Department of Microbiology and Immunology, Weill Cornell Medicine, New York, New York; 2 Department of Radiation Oncology, Weill Cornell Medicine, New York, New York; 3 Department of Urology, Weill Cornell Medicine, New York, New York; 4 Department of Pathology and Laboratory Medicine, Weill Cornell Medicine, New York, New York; 5 Department of Medical Microbiology and Infection Prevention, Amsterdam University Medical Centers, University of Amsterdam, Amsterdam Infection & Immunity Institute, Amsterdam, the Netherlands; 6 Department of Medicine, Weill Cornell Medicine, New York, New York; + TJK and DC made equal contributions to this paper; # Current address: Antibody Research & Technology, Genmab Inc

## Abstract

The approved Pfizer and Moderna mRNA vaccines are well known to induce serum antibody responses to the SARS-CoV-2 Spike (S)-protein. However, their abilities to elicit mucosal immune responses have not been reported. Saliva antibodies represent mucosal responses that may be relevant to how mRNA vaccines prevent oral and nasal SARS-CoV-2 transmission. Here, we describe the outcome of a cross-sectional study on a healthcare worker cohort (WELCOME-NYPH), in which we assessed whether IgM, IgG, and IgA antibodies to the S-protein and its receptor-binding domain (RBD) were present in serum and saliva samples. Anti-S-protein IgG was detected in 14/31 and 66/66 of saliva samples from uninfected participants after vaccine doses-1 and -2, respectively. IgA antibodies to the S-protein were present in 40/66 saliva samples after dose 2. Anti-S-protein IgG was present in every serum sample from recipients of 2 vaccine doses. Vaccine-induced antibodies against the RBD were also frequently present in saliva and sera. These findings may help our understanding of whether and how vaccines may impede SARS-CoV-2 transmission, including to oral cavity target cells.

## INTRODUCTION

Vaccines are critical for curtailing the COVID-19 pandemic [1, 2]. In the United States, 2 highly protective mRNA vaccines are available: BNT162b2 (Pfizer/BioNTech) and mRNA-1273 (Moderna) [3, 4]. These vaccines induce antibodies to the SARS-CoV-2 S-protein, including neutralizing antibodies (NAbs) predominantly directed against the receptor binding domain [1–4]. Serum NAbs are induced at modest levels within ~1 week of dose 1 and strongly boosted by dose 2 at 3 (Pfizer) or 4 weeks (Moderna) [3, 4]. SARS-CoV-2 is typically transmitted nasally or orally and infects cells in the mucosae of the respiratory and gastrointestinal tracts [5–8]. Although serum NAbs are a correlate of protection against COVID-19 [9, 10], mucosal antibodies might directly prevent or limit virus acquisition by the nasal, oral, and conjunctival routes [5–8, 11]. Indeed, antibodies in the respiratory tract or oral cavity have been deemed important to protection against a human-to-human transmitted hantavirus, influenza virus, and respiratory syncytial virus [12–14]. Vaccines based on adenovirus vectors can induce mucosal immunity to the latter 2 viruses [13, 14]. Whether mRNA vaccines induce mucosal immunity requires more study [9]. We report that antibodies to the S-protein are present in saliva samples from vaccinated healthcare workers (HCW). Within 1-2 weeks after their second dose, 53/53 and 13/13 recipients of the Pfizer and Moderna vaccines, respectively, had saliva S-protein IgG antibodies, while IgA was detected in a substantial proportion. These observations may be relevant to vaccine-mediated protection from SARS-CoV-2 infection and disease.

## METHODS

### Patients and Data Sources

The NYP-WELCOME (WEilL COrnell Medicine Employees) trial was initiated on June 16, 2020 at Weill Cornell Medicine (WCM) and New York Presbyterian Hospital (NYP). The aim of the trial is to study the diverse COVID-19 outcomes (from infection to resolution or death and before, during, and after vaccination) among an exposed population in Manhattan, consisting of NYP-Weill Cornell Medicine, asymptomatic, volunteer HCWs. Sequential specimens and questionnaires are collected twice monthly for 3 months and then monthly for 2 years. Samples are collected within the Cornell Clinical & Translational Science Center and stored in the Institutional Biobank. As of 4/1/21, the biobank contains 12,836 trial specimens: PBMCs, saliva, stool, urine, plasma, serum and nasopharyngeal (NP) swab extracts. Here, we used serum and saliva samples only.

NYP-WELCOME inclusion criteria are a completed Informed Consent; age ≥ 18 years old; currently working at Weill Cornell Medicine as a New York Presbyterian or Weill Cornell Medicine employee; able to speak and read English; at risk for COVID-19 through working in 1 or more of the following sites: intensive care unit; emergency department; emergency services; COVID-19 hospital unit/ward; respiratory services; COVID-19 testing location; inpatient hospital unit/area with potential COVID-19 cases; WCM-staffed outpatient area; WCM research laboratory; or by interacting, even occasionally, with WCM clinical or research faculty. Exclusion criteria are a prior diagnosis of COVID-19 infection by a commercially available test; participation in a COVID-19 prophylaxis trial within 30 days of consent; respiratory or gastrointestinal illness with new-onset fever (temperature > 100.4°F); ongoing cough or dyspnea within 14 days; pulse oximeter <94%.

The WCM Institutional Review Board approved the WELCOME trial (Weill Cornell Medicine Employees) on 6.3.2020 with protocol number 20-04021831. Silvia C. Formenti MD is the Principal Investigator. Demographic information on the NYP-WELCOME trial is summarized in the Results section.

### Sample processing

Blood was processed into serum that was heat-inactivated at 56°C. Nonidet-P40 (NP40) non-ionic detergent was added to saliva samples to a final concentration of 0.05% (vol/vol), both to inactivate SARS-CoV-2 and as a preservative [15]. Saliva extracts were sterilely passed through a 22 μm filter, before addition of protease inhibitors to inhibit sample degradation. Inhibitor sources and final concentrations were Aprotinin, 8.5 μg/mL (Sigma Aldrich); phenylmethanesulfonyl fluoride, 5.9mM (Sigma-Aldrich 93482); and sodium orthovanadate, 1.2mM (Sigma-Aldrich S6508). Processed saliva samples were stored at −20°C [16]. In pilot experiments, we confirmed that NP40 and the aforementioned protease inhibitors had no effect on anti-S-protein titers derived using heat-inactivated saliva samples or the CR3022 anti-RBD MAb. The antibody titers were also unaffected when saliva samples were subjected to 3 freeze/thaw cycles.

### S-protein and RBD-protein production

The expression construct for the pre-fusion, S2-P stabilized SARS-CoV-2 S-Foldon-StreptagII S-protein ectodomain was derived as follows. The gene encoding residues 1-1138 from the Wuhan-Hu-1 strain (Genbank MN908947.3) was modified by introducing proline substitutions at residues 986 and 987 and a GGGG-substitution at residues 682-685 (Furin cleavage site). The modified gene was cloned into a pPPI4 plasmid containing a T4 trimerization domain followed by Strep-tag® II [17]. For a 1 L transfection of ExpiCHO cells (6 x 10^6^ per mL), 800 μg of plasmid, 1.6 mL of FectoPRO reagent (Polyplus-transfection SA) and 500 μL of FectoPRO booster were added to 40 mL of Opti-MEM (Thermo Fisher Scientific). Culture supernatants were harvested 3 days post-transfection, centrifuged for 1 hour at 6900g and passed through a 0.2 μm filter (Thermo Fisher Scientific). BioLock-Biotin blocking solution (IBA Lifesciences) was added before the treated supernatants were passed over StrepTactin™ Sepharose resin (GE Healthcare). S-proteins were eluted in 2.5mM desthiobiotin in 100mM Tris-HCl, 150mM NaCl, 1mM EDTA, pH 8.0, dialyzed into PBS and concentrated using Vivaspin protein spin columns with a 100 kDa molecular weight cutoff (GE Healthcare). Protein concentrations were determined using the BCA protein assay kit (Thermo Fisher). Purity was assessed on a 4% to 16%, Bis-Tris Native PAGE gel system (Invitrogen). For SDS-PAGE, purified proteins were denatured with 0.1mM dithiothreitol (DTT) before loading onto a 4% to12% Bis-Tris Gel NuPAGE gel (Invitrogen).

S-proteins from the 229E, HKU1, NL63, and OC43 endemic CoVs were also expressed in ExpiCHO cells and purified using StrepTactin™ Sepharose resin (GE Healthcare), as described above. The HCoV-NL63-S-foldon-StreptagII and HCoV-229E-S-foldon-S-StreptagII plasmids were generated by Philip Brouwer (AMC), Amsterdam, while the HCoV-OC43-S-StreptagII and HCoV-HKU1-S-StreptagII plasmids were provided by Gabriel Ozorowski (Scripps Research Institute, La Jolla).

The SARS-CoV-2-RBD-StrepII expression construct was a gift from Dr. Lakshamanane Prem-kumar (University of North Carolina) and has been described previously [18]. The construct was expressed and purified as described for the S-protein, except that the dialyzed RBD-protein was concentrated using a spin column with a 10 kDa molecular weight cutoff.

### ELISA procedures

The assay to quantify SARS-CoV-2 S-protein antibodies was modified from one described previously [19]. For serum samples, S-proteins (200 ng in 100 μL) were coated overnight onto 96-well plates at 4°C. After 3 washes with PBS/0.05% Tween-20 (PBST), the wells were blocked for 1 hour with 4% (w/vol) powdered milk/PBS (150 μL/well). Serum was initially diluted 1/100 in PBS containing 4% milk and 20% sheep serum, serially diluted as needed, and added to wells for 1 hour. Bound antibodies were detected using goat anti-human horse radish peroxidase (HRP)-conjugated antibodies: anti-IgA from Southern Biotech (2050-05), diluted 1/3000 in 4% milk/PBS; anti-IgG from Jackson ImmunoResearch (109-035-008), diluted 1/5000; and anti-IgM from Jackson ImmunoResearch (109-035-043), diluted 1/5000. After washing, 50 μL of HRP substrate (Thermo Scientific 34029) was added to each well for 3 minutes. Color development was terminated with 0.3 N sulfuric acid, and plates were read at 450 nm using an EnSpire instrument (Perkin Elmer).

To assay saliva samples, modifications were made to increase assay sensitivity and conserve sample volume. The 96-well plates were replaced by 384-well plates (Thermo Scientific 464718). Incubation volumes were correspondingly smaller (10 μL/well) except for the blocking buffer (100 μL/well). The amount of S-protein (SARS-CoV-2 or, when appropriate, from the endemic CoVs) added to wells was 100 ng in 10 μL, to create a higher coating density. The HRP substrate volume was increased to 25 μL/well and the reaction time lengthened to 15 minutes. Accordingly, the colorimetric signals derived from the saliva and serum assays are not directly comparable.

ELISAs to quantify anti-RBD antibodies were performed as described for S-proteins. The RBD-proteins were coated at 100 ng in 100 μL for testing sera and 100 ng in 10 μL for saliva. ELISA-derived net OD450 values for 4-fold dilutions of saliva and 100-fold dilutions of sera were determined by subtracting background values from wells containing no S-protein. The cut-off for positive antibody detection was set to a net OD450 of 0.300. This value corresponds to 6-times the average net OD450 derived from negative samples (324/327 below cut-off, yielding a spec-ificity of 99%). Net OD450 values were plotted longitudinally for each study participant. For 5 vaccinated participants (Pfizer, n = 3; Moderna, n = 2), no saliva sample collected at any time-point could be processed successfully because the ELISA background values (no S-protein) were unacceptably high. Thus, for these samples the OD450 values were > 0.400, which was twice the median background for all samples tested. These 5 participants were excluded from the analyses described here. Twenty additional samples from 13 different participants were also excluded for the same reason, although in each case samples from other timepoints could be processed successfully and were included in the analyses.

To assess the specificity and sensitivity of IgA detection, purified human IgG (Sigma 14506), purified Secretory IgA (SIgA) (BioRad PHP133), or recombinant IgA1 lambda (BioRad HCA172) were coated onto ELISA plates (Corning 3690) and probed with goat anti-Human IgA-HRP (Southern Biotech 2050-05).

## RESULTS

### The NYP-WELCOME trial

The NYP-WELCOME trial is described in the Methods section. To date (4/1/21), 97 participants have completed 605 study visits, with 94 remaining in the trial (3 withdrew at various times). The cohort includes 72 females and 22 males. Of the 94 participants, 61 are white, 9 are Asian, 2 are Black, 18 self-reported as “Other”, and 4 declined to provide information. The median age of cohort members is 39.5 years.

Seven participants were SARS-CoV-2-infected before joining the trial. Two more became infected during the trial, as determined by a positive NP swab RT-PCR test, and 1 more as judged by positivity for serum S-protein antibodies at multiple time-points. Of the 10 infected people, 8 were later vaccinated.

Pfizer and Moderna mRNA vaccines became available at NYP-WCM after December 15th, 2020. As of 4/1/21, 85/94 NYP-WELCOME participants have received at least 1 mRNA vaccine dose (Pfizer, n = 68 participants; Moderna, n = 17). One male participant received an experimental vaccine candidate (Novavax) or a placebo, at which point he was excluded from this study. Of 85 mRNA vaccine recipients, 66 were female and 19 male. As outlined below, we successfully detected S-protein antibodies in saliva and serum samples from both 55 Pfizer and 14 Moderna uninfected vaccine recipients. Among these 69 individuals, samples were available from 66 after 2 doses while 2 Pfizer and 1 Moderna vaccine recipients provided samples only after the first dose.

### Detection of antibodies to the SARS-CoV-2 S-protein and RBD in saliva and serum

**Figure 1.**
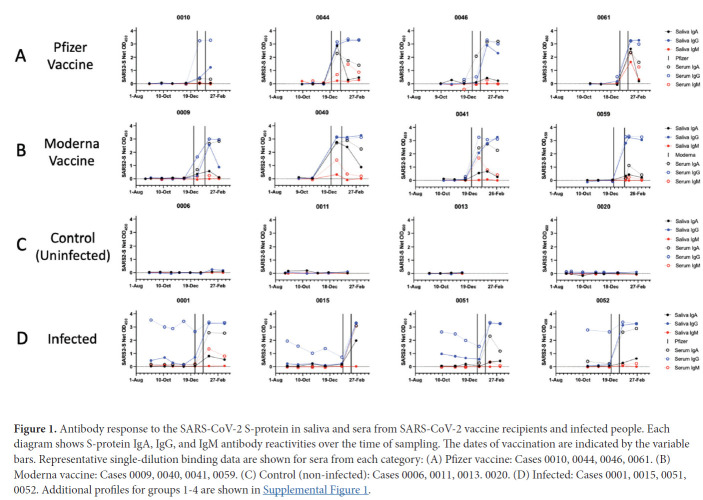
Antibody response to the SARS-CoV-2 S-protein in saliva and sera from SARS-CoV-2 vaccine recipients and infected people. Each diagram shows S-protein IgA, IgG, and IgM antibody reactivities over the time of sampling. The dates of vaccination are indicated by the variable bars. Representative single-dilution binding data are shown for sera from each category: (A) Pfizer vaccine: Cases 0010, 0044, 0046, 0061. (B) Moderna vaccine: Cases 0009, 0040, 0041, 0059. (C) Control (non-infected): Cases 0006, 0011, 0013. 0020. (D) Infected: Cases 0001, 0015, 0051, 0052. Additional profiles for groups 1-4 are shown in Supplemental Figure 1.

**Figure 2.**
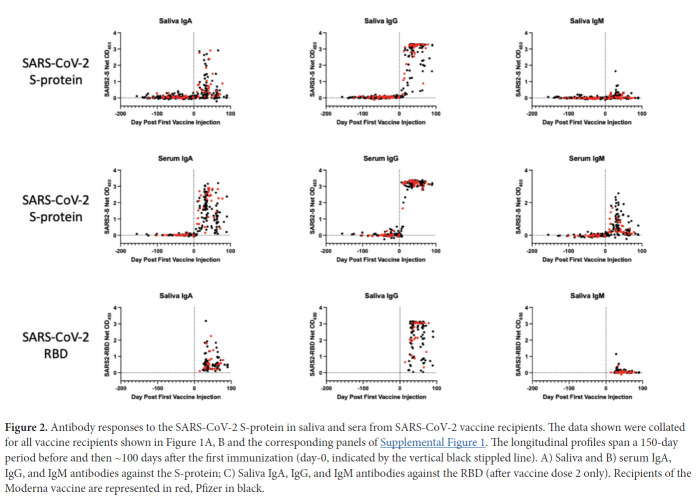
Antibody responses to the SARS-CoV-2 S-protein in saliva and sera from SARS-CoV-2 vaccine recipients. The data shown were collated for all vaccine recipients shown in Figure 1A, B and the corresponding panels of Supplemental Figure 1. The longitudinal profiles span a 150-day period before and then ~100 days after the first immunization (day-0, indicated by the vertical black stippled line). A) Saliva and B) serum IgA, IgG, and IgM antibodies against the S-protein; C) Saliva IgA, IgG, and IgM antibodies against the RBD (after vaccine dose 2 only). Recipients of the Moderna vaccine are represented in red, Pfizer in black.

We assessed the development of antibody responses to the SARS-CoV-2 S-protein in serum and saliva samples from mRNA-vaccinated HCWs. We were able to process saliva samples successfully from the majority of trial participants (Table 1) and performed antibody assays on sera from the same subset of individuals, for comparison. Longitudinal profiles for S-protein IgA, IgG, and IgM saliva and serum responses in selected individuals from Groups 1-4 are shown in Figure 1. A collated data set for Groups 1 and 2 is presented in Figure 2, which also includes data on saliva antibody responses to the SARS-CoV-2 RBD-protein (Figure 2C). The profiles selected for display in Figure 1 are those for which sample collection dates were well matched to when the vaccines were administered. Additional longitudinal profiles are shown as Supplemental Figure 1. Recipients of the Pfizer and Moderna vaccines form Groups 1 and 2, respectively (Figure 1A, B; Supplemental Figure 1A, B). For comparison, we also studied 2 other sub-groups of participants. Group-3 members were SARS-CoV-2 uninfected (n = 7) (Figure 1C, Supplemental Figure 1C), while Group 4 included people who had recovered from COVID-19 or became infected during participation in the NYP-WELCOME trial (n = 10). Among Group-4 members, 8 were vaccinated (Figure 1D, Supplemental Figure 1D).

**Table 1.** Saliva samples successfully processed from NYP-WELCOME cohort members

	Number vaccinated	Saliva samples analyzed between dose 1 and dose 2	Saliva samples analyzed after dose 2
Group 1 Pfizer Vaccine	55	24	53
Group 2 Moderna Vaccine	14	7	13

The net OD_450_ values from the ELISAs were derived from saliva diluted 1/4 and sera diluted 1/100. This difference together with others described in Methods mean that the signals from the saliva and serum ELISAs are not directly comparable. The saliva assay is the more sensitive of the 2 (see Figure 5). Note that antibody concentrations in saliva are generally much lower than in sera (see Figure 5). Differences in coating concentrations also affect the direct comparability of assays performed using the SARS-CoV-2 S-protein and its RBD.

**Figure 3.**
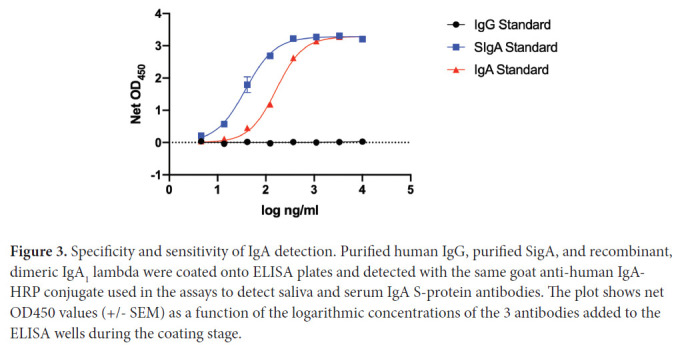
Specificity and sensitivity of IgA detection. Purified human IgG, purified SigA, and recombinant, dimeric IgA_1_ lambda were coated onto ELISA plates and detected with the same goat anti-human IgA HRP conjugate used in the assays to detect saliva and serum IgA S-protein antibodies. The plot shows net OD450 values (+/− SEM) as a function of the logarithmic concentrations of the 3 antibodies added to the ELISA wells during the coating stage.

The IgA-detection conjugate is specific for IgA and does not cross-react with IgG (Figure 3). Both SIgA and recombinant, dimeric IgA standards are detected, but for simplicity we routinely refer to what we have assayed as being IgA. The ~5-fold more sensitive detection of SigA could arise from coating efficiency differences vs IgA under the ELISA conditions used and/or conjugate properties (Figure 3).

The NYP-WELCOME cohort was not established as a vaccine study, so there was no coordination between the dates of vaccine administration and the approximately monthly sample collection dates. Hence, samples were not available for some participants in the 3-(Pfizer) and 4-week (Moderna) period between the 2 vaccine doses (Table 1). Overall, however, sufficient samples were available from before and after each vaccination to allow conclusions to be drawn.

The longitudinal profiles (Figures 1A, B; Supplemental Figure 1A, B) and collated data (Figure 2, Table 2) show that the Pfizer and Moderna vaccines rapidly and consistently induce S-protein-specific IgG and IgA in both saliva and sera, with IgM occasionally detected. The proportions of vaccinated individuals with saliva and serum IgG, IgA, and IgM S-protein antibodies after the first and second doses are summarized in Table 2. Antibody responses against the SARS-CoV-2 RBD-protein were also determined for all Group 1 and Group 2 samples collected after the second vaccine dose (Figure 2C, Table 2).

**Table 2.** Proportions of vaccinated individuals with saliva and serum IgG, IgM, and IgA S-protein antibodies after the first and second doses

Group 1: Pfizer vaccine						
	IgA		IgG		IgM	
	Saliva	Serum	Saliva	Serum	Saliva	Serum
S-protein After dose 1	4/24 (17%)	9/24 (38%)	8/24 (33%)	13/24 (54%)	0/24 (0%)	4/24 (17%)
S-protein After dose 2	29/53 (55%)	52/52 (100%)	53/53 (100%)	52/52 (100%)	9/53 (17%)	37/52 (71%)
RBD After dose 2	44/53 (83%)	39/51 (76%)	53/53 (100%)	51/51 (100%)	2/53 (4%)	28/51 (55%)

Group 2: Moderna vaccine						
	IgA		IgG		IgM	
	Saliva	Serum	Saliva	Serum	Saliva	Serum
S-protein After dose 1	5/7 (71%)	7/7 (100%)	6/7 (86%)	7/7 (100%)	1/7 (14%)	5/7 (71%)
S-protein After dose 2	11/13 (85%)	13/13 (100%)	13/13 (100%)	13/13 (100%)	1/13 (8%)	8/13 (62%)
RBD After dose 2	10/13 (77%)	13/13 (100%)	13/13 (100%)	13/13 (100%)	1/13 (8%)	6/13 (46%)

The denominator values for saliva in this table are those recorded in Table 1. The antigen used to detect the antibodies (S-protein or RBD) is indicated. One serum sample from the Pfizer vaccine group was not available.

Saliva samples from 31 individuals at various times during the inter-dose period were successfully processed, with S-protein IgG detectable in 14 of them during this period (Figure 1A, B; Supplemental Figure 1A, B, Table 2). Two Pfizer and 1 Moderna vaccine recipients provided saliva samples after dose 1 but had not received their second dose (Supplemental Figure 1A, B). After dose 2, 66 vaccine recipients (53 Pfizer, 13 Moderna) were positive for saliva S-protein IgG, with IgA antibodies frequently but not uniformly detected and IgM present only rarely. All vaccine recipients had serum IgG and IgA antibodies to the S-protein after dose 2, and most also had IgM. Anti-RBD IgG antibodies were detected in 100% of saliva and serum samples after dose 2, while IgA was detected frequently and IgM occasionally (Figure 2C, Table 2).

In contrast to the response to vaccination, S-protein antibodies were not detectable in saliva samples from uninfected control individuals (Figure 1C, Supplemental Figure 1C). Trial participants who became virus-infected before or during the trial did, however, have S-protein IgG in their saliva, although these infection-elicited responses tended to wane over a multi-month period. IgA antibodies were occasionally detected at lower levels in these saliva samples (Figure 1D, Supplemental Figure 1D). Observations of S-protein IgG and IgA in saliva from SARS-CoV-2 infected people are consistent with previous reports [5–8, 20–22]. When 8 infected individuals (0001, 0014, 0015, 0037, 0051, 0052, 0053, and 0074) were vaccinated, their saliva and serum S-protein IgA and IgG reactivities and, in 1 case also IgM, were rapidly boosted (Figure 1D and Supplemental Figure 1D).

### S-protein IgA in saliva from an uninfected individual, pre-vaccination

**Figure 4.**
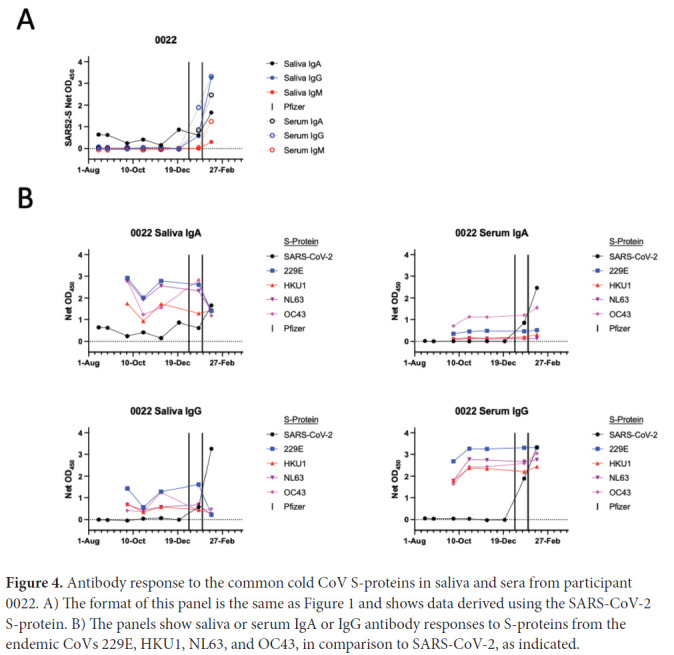
Antibody response to the common cold CoV S-proteins in saliva and sera from participant 0022. A) The format of this panel is the same as Figure 1 and shows data derived using the SARS-CoV-2 S-protein. B) The panels show saliva or serum IgA or IgG antibody responses to S-proteins from the endemic CoVs 229E, HKU1, NL63, and OC43, in comparison to SARS-CoV-2, as indicated.

Pre-vaccination saliva samples from one participant, 0022, who had no documented evidence of SARS-CoV-2 infection and was seronegative for S-protein antibodies, consistently contained S-protein IgA but not IgG. After vaccination, saliva IgA and IgG levels both increased in participant 0022 (Figure 4A). One explanation is that the saliva IgA against the SARS-CoV-2 S-protein may reflect virus exposure that did not lead to systemic infection but was sufficient to trigger a mucosal immune response. There are reports of mucosal anti-S-protein IgA in uninfected, seronegative individuals [23, 24]. However, we explored an alternative explanation by testing the 0022 saliva and serum samples against S-proteins from the 229E, HKU1, NL63, and OC43 viruses (Figure 4B). IgAs against all 4 of these CoV S-proteins were detected in saliva, as were the corresponding serum IgGs. Anti-S-protein saliva IgG and serum IgA to the endemic CoVs were also observed although less consistently (Figure 4B). A cross-reactive antibody response to an endemic CoV infection seems the more likely explanation for the saliva IgA detected in participant 0022 using the SARS-CoV-2 S-protein (Figure 4A, B).

### Comparative magnitude of anti-S-protein responses in saliva and sera

**Figure 5.**
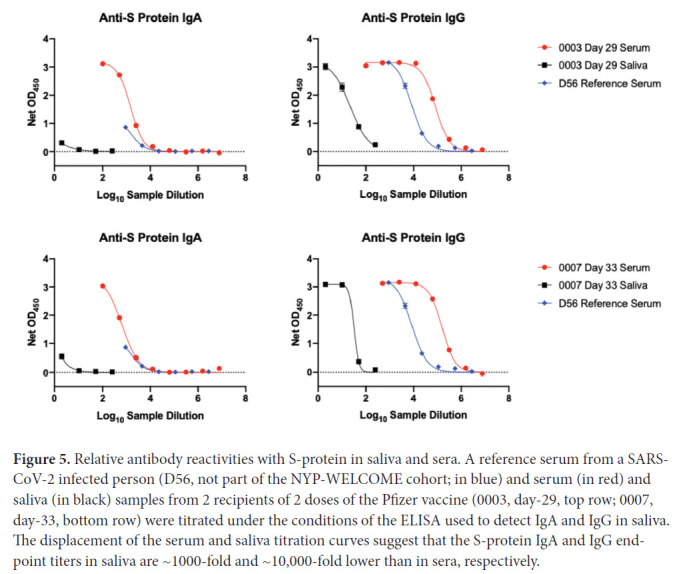
Relative antibody reactivities with S-protein in saliva and sera. A reference serum from a SARS-CoV-2 infected person (D56, not part of the NYP-WELCOME cohort; in blue) and serum (in red) and saliva (in black) samples from 2 recipients of 2 doses of the Pfizer vaccine (0003, day-29, top row; 0007, day-33, bottom row) were titrated under the conditions of the ELISA used to detect IgA and IgG in saliva. The displacement of the serum and saliva titration curves suggest that the S-protein IgA and IgG end-point titers in saliva are ~1000-fold and ~10,000-fold lower than in sera, respectively.

The data for saliva and serum S-protein antibodies in Figures 1 and 2 are derived from ELISAs performed under different conditions (S-protein coating amounts and sample dilutions; see Methods section). The saliva assay is the more sensitive of the 2, and hence the data plots should not be interpreted as indicating there are quantitatively similar IgA and IgG reactivities in the 2 fluids. To gain an insight into the relative magnitudes of the saliva and serum responses, we titrated a serum sample from an infected individual (D56) and sera from 2 participants who had each received 2 Pfizer vaccine doses (Cases 0003 and 0007), using the saliva ELISA format, alongside saliva samples from the same individuals (Figure 3). Judged by the displacements of the titration curves, we estimate that the end-point titers of S-protein IgA and IgG antibodies present in saliva are ~1000-fold and ~10,000 fold lower, respectively, than in serum (Figure 5). Note that infection serum D56 neutralized SARS-CoV-2 with an ID_50_ titer of 900 in our SARS-CoV-2 pseudovirus-based neutralization assay [17]. The much lower S-protein antibody reactivities present in saliva are below the detection limit for that assay. However, we consistently detected IgA antibodies to the SARS-CoV-2 RBD in saliva samples after the second vaccine dose (Figure 2C, Table 2). These RBD-reactive antibodies may be capable of virus neutralization.

## DISCUSSION

We show that mRNA vaccines induce not just systemic serum anti-S-protein responses but also IgG and, to a lesser extent, IgA antibodies that are detectable in saliva. The assay we used recognizes dimeric IgA with and without the J-chain and secretory component; ie, SIgA + dIgA (Figure 3). Antibodies in saliva are known to be dominated by SIgA and IgG. Local plasma cells in the stroma of salivary glands secrete dIgA, which traffics through mucosae by interacting with the polymeric Ig receptor. Typically, ~95% of salivary IgA is in the SIgA form. In contrast, IgG in saliva largely originates from plasma, by transudation from the gingival blood circulation [25–27]. IgM, also derived from plasma, is found at lower levels. In healthy people, the total concentrations in saliva are ~150 μg/ml for SIgA, ~15 μg/ml for IgG, and ~5 μg/ml for IgM. In contrast, serum concentrations are ~1000-fold higher for IgG and ~100-fold for total IgA [25, 27]. Because saliva IgG is mostly transudated from blood, it is probable that the anti-S-protein content of saliva largely reflects what is present in serum, albeit in lower amounts. The limited saliva volumes that were collected from the vaccine recipients precluded a more detailed investigation of what IgA forms contribute to the overall saliva anti-S response.

Could vaccine- or infection-induced salivary and, by extension, nasopharyngeal antibodies prevent or limit SARS-CoV-2 infection at the principal portals of entry–the mouth and nose? Our data raise that possibility but cannot provide a definitive answer. Additional studies in this area are justified, particularly now that key target cells for SARS-CoV-2 infection in the oral cavity have been identified [5]. A report on SARS-CoV-2 infection in rhesus macaques shows that virus can still replicate in nasal turbinates even when it is suppressed in the lungs by passively transferred NAbs [28]. Assays of vaccine-induced antibodies extracted from NP swabs might provide useful information, although Ig concentrations in tissue sites, eg, intra-mucosal interstitial fluids, may be impossible to measure by swab-sampling and extraction.

There is increasing evidence that SARS-CoV-2 transmission is prevented by vaccination, quite plausibly by vaccine-elicited NAbs [4, 9, 10, 29, 30]. Only a subset of antibodies to the S-protein is capable of virus neutralization, principally by binding to epitopes in the RBD and the N-terminal domain [31, 32]. Our S-protein ELISA detects both NAbs and non-neutralizing antibodies, and the latter are likely to play little or no role in limiting virus transmission. We did, however, consistently detect antibodies to the RBD in saliva after the second vaccine dose, which suggests that saliva at least has the potential for virus neutralization [31, 32]. We cannot confirm this supposition directly, because we have found that saliva components interfere with pseudovirus-based neutralization assays, and hence that it is necessary to purify the Ig fraction. Unfortunately, the saliva volumes collected in the NYP-WELCOME study are much too low for Ig purification to be practical (the protocol was designed in the summer of 2020, well before the present sub-project was contemplated). IgA in saliva from some infected people can neutralize SARS-CoV-2, and neutralizing IgA persists longer in saliva than serum [3]. Of note also is that RBD-specific IgA reactivities in saliva correlated better with the extent of neutralization than the corresponding saliva IgG reactivities, and that RBD-specific IgA reactivities were higher in saliva than serum [21]. Thus, although we found much lower levels of S-protein antibodies in saliva than in sera (Figure 3), what is present in saliva may be capable of neutralizing at least some of the incoming virus.

There are reports of uninfected, seronegative HCWs whose mucosal samples (tears, nasal swabs, or saliva) contained anti-S-protein IgA [23, 24]. We identified a similar seronegative individual, 0022, whose saliva contained IgA but, notably, not IgG antibodies to the SARS-CoV-2 S-protein. We also detected antibodies to S-proteins from endemic CoVs (Figure 4). It has been argued that these unusual cases may arise when SARS-CoV-2 exposure did not lead to systemic infection but was sufficient to trigger a mucosal response [1]. T cells reactive with SARS-CoV-2 antigens in some highly exposed but uninfected people have also been reported [8, 24]. However, an alternative explanation is that cross-reactivities caused by infections with 1 or more endemic CoVs are responsible. Our data are not sufficient to resolve the uncertainty.

In future studies, it should be possible to determine whether other vaccine designs, such as the Johnson & Johnson and AstraZeneca adenovirus vectors and the Novavax adjuvanted protein, also induce saliva antibodies, and if so to what extent. Comparing the efficacies of different vaccines at preventing transmission to their abilities to induce mucosal antibody responses may be valuable. For example, a meta-analysis of HIV-1 Env vaccine trials showed that adjuvanted proteins induced mucosal IgG antibodies more consistently than DNA or virus vector vaccines, while IgA antibodies to Env were rarely elicited at mucosal sites by any of the immunogens evaluated [33]).

In conclusion, antibodies against the SARS-CoV-2 S-protein and its RBD are present in every saliva sample from HCWs given 2 doses of Pfizer or Moderna mRNA COVID-19 vaccines. Both IgG and IgA were detected, the former probably representing antibodies transudated from the blood into mucosal sites. While antibodies are present in saliva at much lower levels than serum, it is possible they play a role in preventing or limiting infection when SARS-CoV-2 is transmitted via the nose and mouth. Given the preponderance of these routes in establishing new infections, knowledge of localized antibody responses to vaccination may help us understand their protective mechanisms.

## SUPPLEMENTARY DATA

Supplementary materials are available at the *Pathogens and Immunity* website. Supplementary data may be provided by the authors to benefit the reader. Supplementary data are not copyedited and are the sole responsibility of the authors. Questions or comments related to supplementary materials should be addressed to the corresponding author.
