# Supplementary material for: Antibody Responses to SARS-CoV-2 mRNA Vaccines Are Detectable in Saliva
Source: Pathog Immun. 2021 Jun 7;6(1):116–34. doi: 10.20411/pai.v6i1.441 (PMC8201795; doi:10.20411/pai.v6i1.441)
Supplement: Supplemental Figure 1 [file pai-6-116-s01.pdf]

Supplemental Information

Supplemental Figure 1. Antibody response to the SARS-CoV-2 S-protein in saliva and sera from SARS-CoV-2 vaccine recipients and infected people. The format of this figure is the same as Figure 1 and shows additional longitudinal profiles for trial participants in Groups 1-4, as indicated.

A

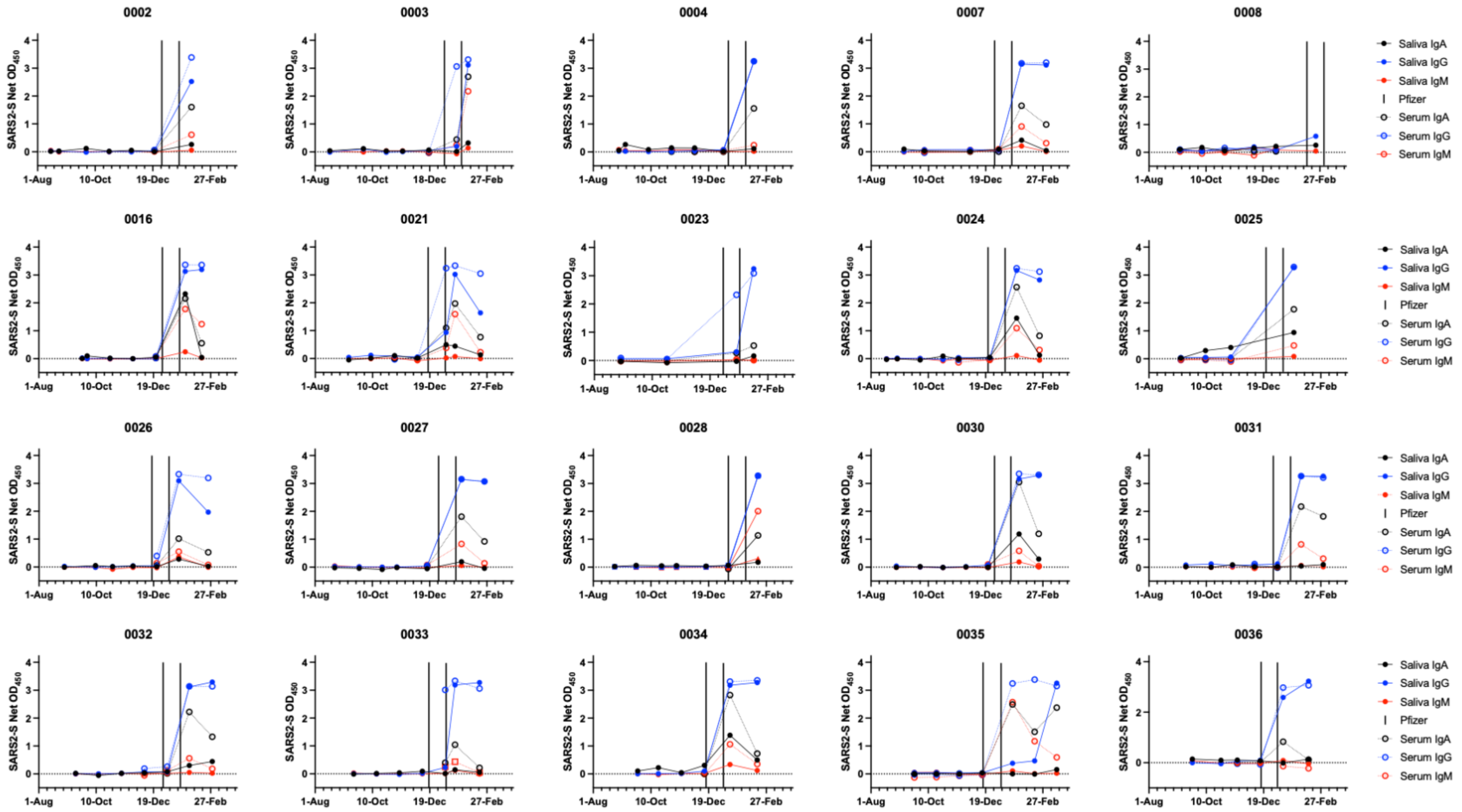
0002
0003
0004
0007
0008
0016
0021
0023
0024
0025
0026
0027
0028
0030
0031
0032
0033
0034
0035
0036
SARS2-S Net OD$_{450}$
SARS2-S OD$_{450}$
1-Aug
10-Oct
18-Dec
19-Dec
27-Feb
Saliva IgA
Saliva IgG
Saliva IgM
Pfizer
Serum IgA
Serum IgG
Serum IgM

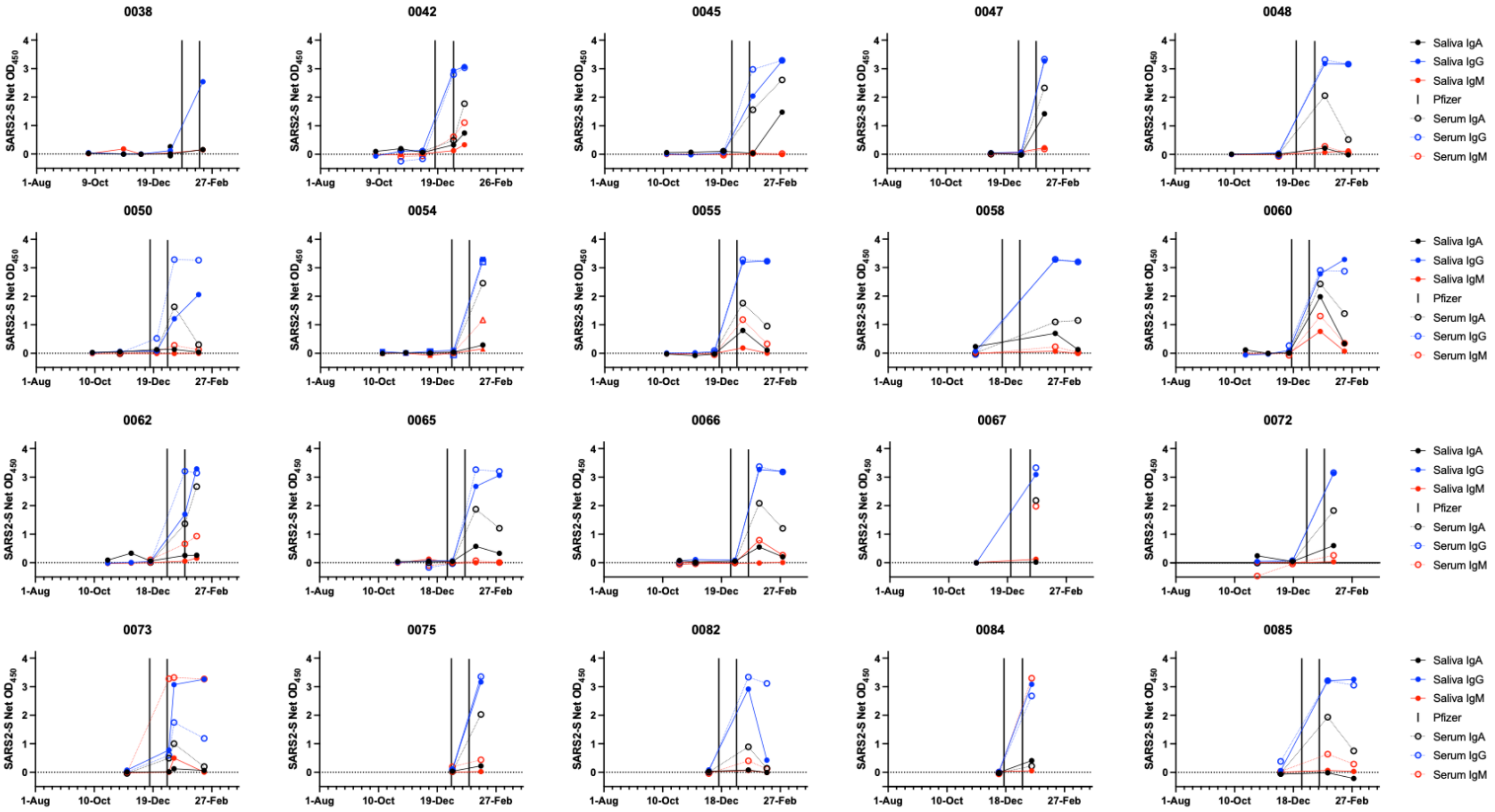
0038
0042
0045
0047
0048
0050
0054
0055
0058
0060
0062
0065
0066
0067
0072
0073
0075
0082
0084
0085
SARS2-S Net OD450
1-Aug
9-Oct
10-Oct
18-Dec
19-Dec
26-Feb
27-Feb
Saliva IgA
Saliva IgG
Saliva IgM
Pfizer
Serum IgA
Serum IgG
Serum IgM

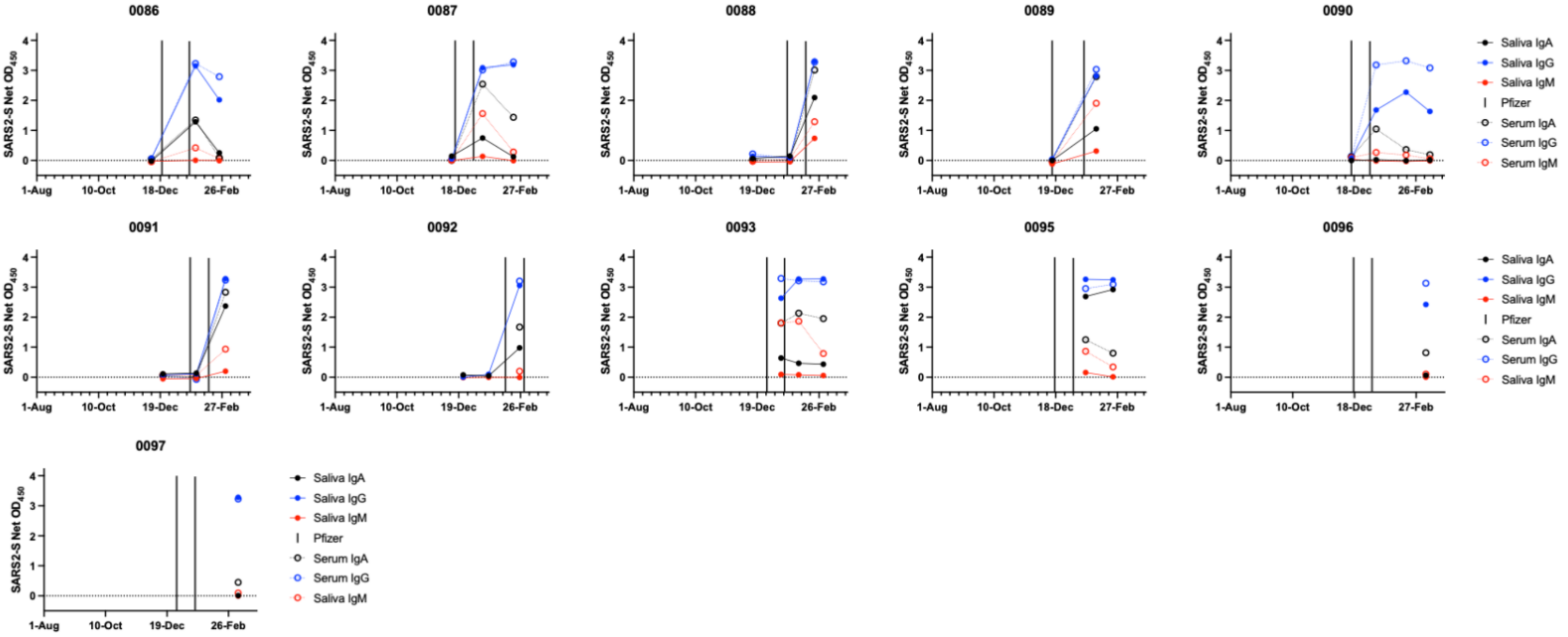
0086
0087
0088
0089
0090
0091
0092
0093
0095
0096
0097
SARS2-S Net OD_{450}
1-Aug
10-Oct
18-Dec
19-Dec
26-Feb
27-Feb
Saliva IgA
Saliva IgG
Saliva IgM
Pfizer
Serum IgA
Serum IgG
Serum IgM
Saliva IgM

B

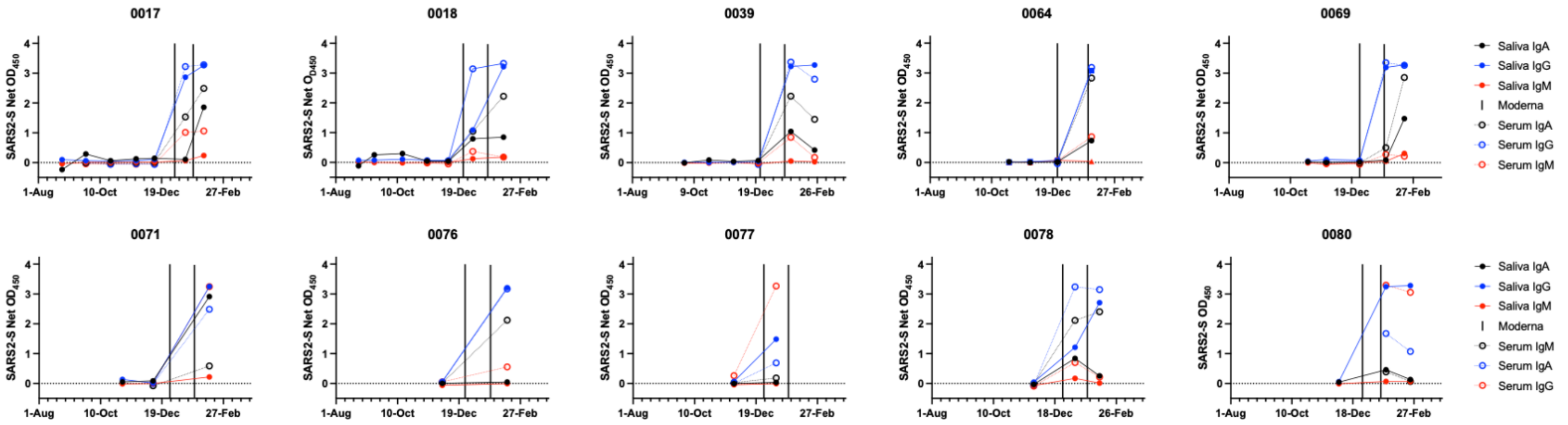
0017
0018
0039
0064
0069
0071
0076
0077
0078
0080
SARS2-S Net OD450
SARS2-S Net OD450
SARS2-S OD450
1-Aug
10-Oct
19-Dec
27-Feb
9-Oct
26-Feb
18-Dec
Saliva IgA
Saliva IgG
Saliva IgM
Moderna
Serum IgA
Serum IgG
Serum IgM
Saliva IgA
Saliva IgG
Saliva IgM
Moderna
Serum IgM
Serum IgA
Serum IgG

C

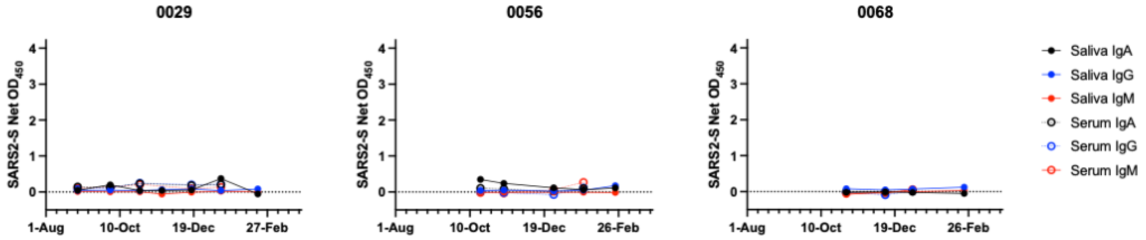
0029
0056
0068
SARS2-S Net OD450
1-Aug
10-Oct
19-Dec
27-Feb
26-Feb
Saliva IgA
Saliva IgG
Saliva IgM
Serum IgA
Serum IgG
Serum IgM

D

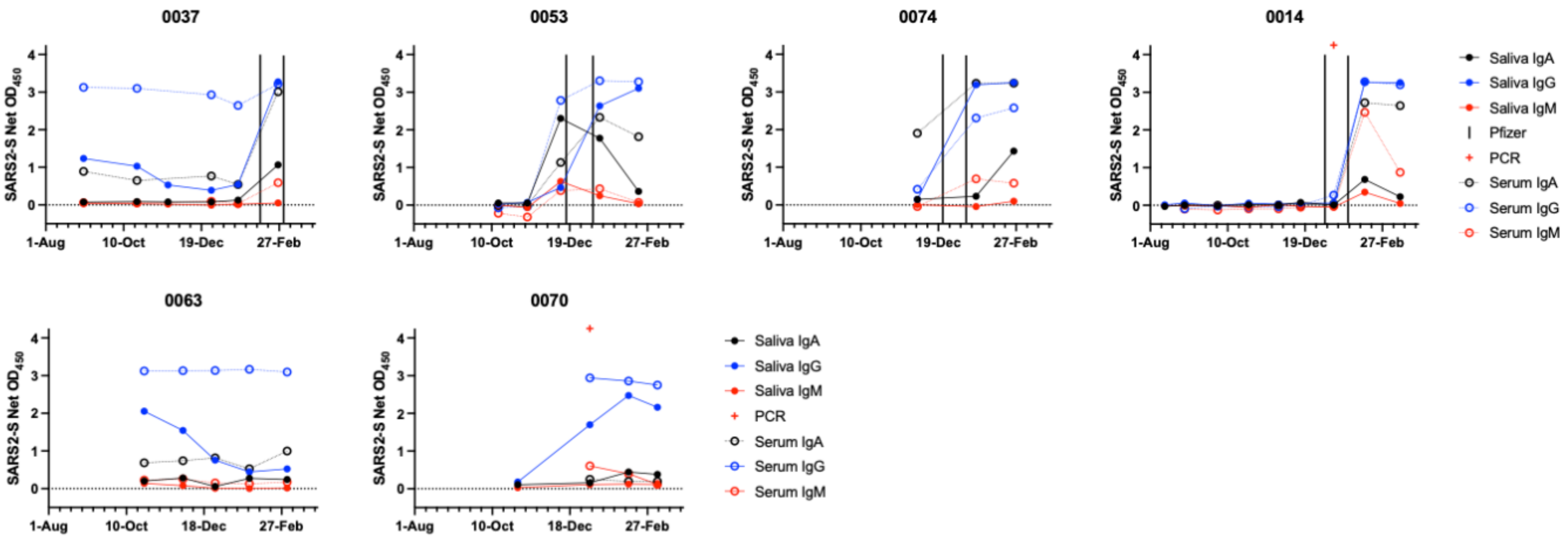
0037
0053
0074
0014
0063
0070
SARS2-S Net OD450
1-Aug
10-Oct
19-Dec
18-Dec
27-Feb
Saliva IgA
Saliva IgG
Saliva IgM
Pfizer
PCR
Serum IgA
Serum IgG
Serum IgM
